# Dying for a Drink: An Alcohol Care Team Evaluation Two Years Post Implementation

**DOI:** 10.1192/bjo.2025.10483

**Published:** 2025-06-20

**Authors:** Katie French, Denise Garton

**Affiliations:** Derbyshire Healthcare NHS Foundation Trust, Derby, United Kingdom

## Abstract

**Aims:** Alcohol specific mortality and alcohol-related hospital admissions in England have continued to rise, with local statistics for Derbyshire worse than the national average. Alcohol Care Teams (ACTs) aim to improve the care received by those in hospital for alcohol misuse, with evidence showing they can reduce admissions, readmissions, and length of stay. Since ACTs have been stipulated in the NHS long-term plan, we wanted to gain insight into our local provision at the Royal Derby Hospital and the impact of the service two years post implementation.

**Methods:** Data relating to presentations, care provision, and outcomes were extracted from the electronic patient record system from September 2022 to August 2024. 3514 adults aged 18 years and over were referred to the ACT. Data regarding hospital admission rates, readmission rates, length of stay, and admission codes were requested from the University Hospitals of Derby and Burton Trust.

**Results:** Alcohol referral numbers have increased by 58%, with 76% of patients receiving an assessment or advice and guidance. For those not seen, 70% were discharged before an intervention could take place. 49% of patients had an AUDIT-C (Alcohol Use Disorders Identification Test for Consumption) score of 12, with scores over 10 indicating potential alcohol dependency and significant complexities. Alcohol-related readmissions have decreased with an overall reduction of 16.67% post-implementation despite an increase in readmissions across all hospital presentations. Length of stay for these patients increased from 6.56 days to 7.39 days in year one but dropped to 5.93 days in year two. 37.5% of referrals to the ACT were referred to, encouraged to self-refer to or already under the care of community alcohol services and 21% of patients were offered a FibroScan appointment with the ACT.

**Conclusion:** Based on local estimates of a readmission costing £2000, the service has demonstrated savings of £1.14 million in year 1 and £448,000 in year 2. Length of stay data for hospital admissions increased in year 1, but we suggest that this may be a proxy for poorer quality of care prior to the ACT, resulting in higher readmission rates. Despite the team evidently operating at their ceiling of capacity with clear unmet need remaining, the evaluation shows the success of our ACT. The service has improved the care offer for patients and has contributed to the reduction of the burden of disease within the hospital, positively impacting the wider system and providing evidence for the efficacy of these services.

